# Furry Animal Allergy: Lipocalins, Serum Albumins, and Secretoglobins—Cross-Reactivity, Diagnosis, and Management Strategies

**DOI:** 10.1007/s12016-025-09086-7

**Published:** 2025-07-30

**Authors:** Weronika Gromek, Natalia Kołdej, Marcin Kurowski, Emilia Majsiak

**Affiliations:** 1 Polish-Ukrainian Foundation of Medicine Development, Nałęczowska 14, 20-701 Lublin, Poland; 2 Student Scientific Association for Allergy, Asthma, and Immunology at the Department of Immunology, Rheumatology, and Allergy Clinic, Medical University of Lodz, Lodz, 90-419 Poland; 3 Department of Immunology and Allergy, Medical University of Lodz, Lodz, 90-419 Poland; 4 Department of Health Promotion, Faculty of Health of Sciences, Medical University of Lublin, Staszica 4/6, Lublin, 20-081 Poland

**Keywords:** Allergy, Furry animals, Molecular allergy diagnostics, Cross reactive determinants (CRD)

## Abstract

Furry animals are familiar companions in modern society. Despite multiple beneficial roles in economic and social contexts, they can be the source of allergenic compounds. Moreover, research indicates that these allergens could be detected even in households where animals are not present. Consequently, the risk of asthma exacerbation is increased. Furthermore, accurately diagnosing a genuine allergy to furry animals remains a significant challenge for medical practitioners. Therefore, this review aims to gather and summarize valid information regarding three main groups of allergens associated with furry animals, including lipocalins, serum albumins, and secretoglobins. In this manuscript, we clarify the molecular structure of allergens, discuss cross-reactions between them, and highlight their clinical importance. We also outline the diagnostic techniques for furry animal allergy, as well as novel, emerging therapies. Additionally, we discuss the occupational risks of allergies for both laboratory workers and cattle farmers.

**Graphical abstract:**

Created in BioRender[1] 
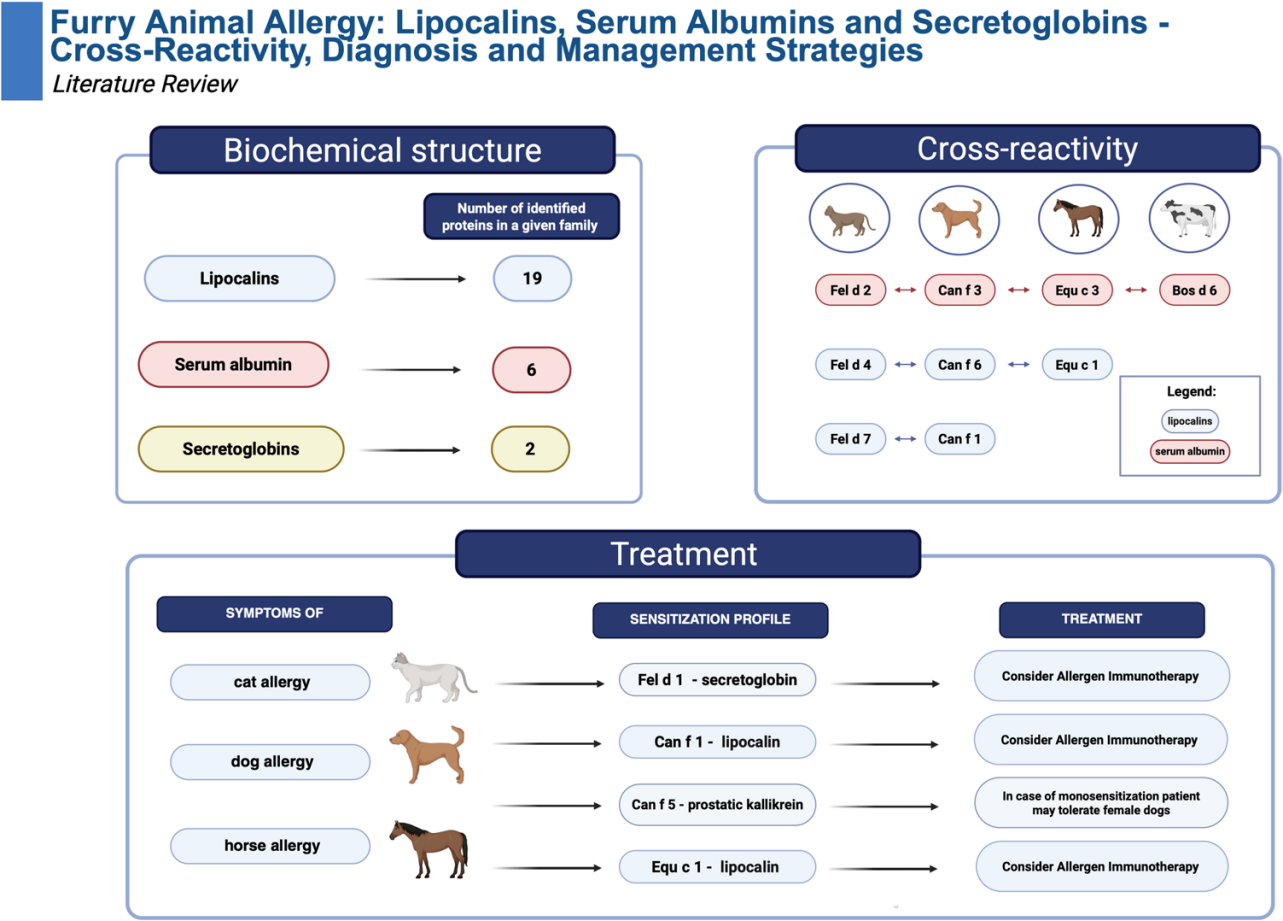

## Introduction

Fifteen thousand years ago, humans began domesticating dogs, which laid the foundation for the domestication of other animal species. As a result, the types of tamed animals vary across different regions of the world [2]. In modern society, companion animals, such as dogs, cats, rabbits, and other pets, have become an integral part of human life. According to the 2023 European Pet Food Industry report, 46% of households in Europe own at least one pet, with cats and dogs being the predominant choice [3]. They are the leading causes of animal allergies, affecting up to 10–20% of people [4].

Research conducted by Dávila et al. demonstrated that 26% of European adults presenting at medical facilities due to suspected allergic reactions to inhaled allergens exhibit sensitivity to cats, while 27% display sensitization to canines [5]. As measured by specific immunoglobulin E (sIgE), the prevalence of sensitization to felines varies globally from 8.6 to 54.4%; in comparison, the occurrence of sensitization to dogs ranges from 6.10 to 64.70% [6, 7]. People with pet allergies manifest diverse symptoms primarily impacting the respiratory system and skin [8].

Furry animals are one of the most considerable sources of year-round indoor inhalant allergens [8]. Close contact with animals may extend beyond domestic environments, incorporating recreational pursuits such as equestrian activities. Additionally, specific individuals may encounter animals as part of their professional responsibilities. Professional groups exposed to potential occupational hazards include laboratory specialists who interact with rodents, zoological garden employees who manage various exotic species, veterinarians, and agricultural workers who are exposed to cattle.

One of the primary diagnostic tools for furry animal allergies (FAA) was, until recently, skin testing. In the 1960 s, immunoglobulin E (IgE) was discovered, which helped to identify its concentration in allergen extracts [9, 10]. In recent years, considerable advancements in IgE assessment methods have been made. Today, we have reached a threshold that allows us to precisely analyze individual allergen molecules. Nowadays, we have diagnostic tests that simultaneously evaluate many allergenic proteins; these tools help physicians in their daily practice to identify the protein that induces the allergic reaction in a patient. Such an achievement helps distinguish genuine allergy cases from cross-reaction and co-sensitization. The utilization of molecular allergy diagnostics can be helpful in this regard. Physicians’ understanding of families of allergenic proteins and their clinical significance is crucial to enhancing care levels for patients with FAA. So far, the furry animal-derived proteins have been identified as belonging to the following families: lipocalins, secretoglobulins, serum albumins, latherin, cystatins, and others. Thanks to testing molecular allergen-specific IgE in clinical practice, we can precisely identify a sensitization to a specific animal, distinguishing between genuine sensitization and cross-sensitization, and qualify individuals for immunotherapy.

Since guidelines for diagnosing FAA are often restricted to the three most common mammals—dogs, cats, and horses—diagnosing allergies to other furry animals can present challenges for doctors in making treatment decisions [11–13]. Therefore, the key objective of this review was to gather information about three of the most important allergen groups of furry animals (lipocalins, serum albumins, and secretoglobins) to facilitate understanding of their clinical relevance. Additionally, we discussed the occupational risks of allergies for laboratory workers and cattle farmers.

## Lipocalins

Lipocalins are a diverse family of proteins widely present in animals, plants, and bacteria [14]. Lipocalins are the predominant mammalian allergenic protein group, constituting approximately 50% of all known allergens in furry animals [15]. They are relatively small particles classified within the calycin superfamily [16, 17]. Their sequence length ranges from 160 to 230 amino acids, with an average estimated molecular size of approximately 17–30 kDa [18–20]. Lipocalins share a highly similar three-dimensional structure but exhibit significant divergence in their amino acid sequences (from 14 to 67) [21, 22] They display a shared tertiary structure consisting of a central β-barrel constructed by eight anti-parallel β-strand*s* [23]. Niemi et al. demonstrated that lipocalins exhibit reduced allergenic potential when present as monomers in solution, possessing a decreased capacity to elicit the secretion of inflammatory mediators in contrast to their dimeric counterparts [24]. Their structure facilitates the transport of hydrophobic molecules. Moreover, they play a significant role in chemical signaling, olfaction, and gustation [22, 25–27]. Lipocalins are recognized as proteins that are exceptionally stable against thermal and chemical denaturation [28]. These physicochemical properties can explain their ability to persist in the environment. They easily adhere to clothes and furniture and, therefore, can be transported over extensive distances. They have been detected in homes, classrooms, cars, and daycare centers, where animals are not typically present [29, 30]. In animals, lipocalins can be produced in various glands and tissues, including salivary glands, tongue, sweat glands, mammary glands, and liver. The principal sources of lipocalins are animals’ saliva, hair, dander, skin, and milk [15]. Differences in lipocalin production have been observed between different species or even within the same species, particularly between sexes. The amount of lipocalins produced differs notably, particularly in rodents [31, 32].

Researchers have found that human tear lipocalin (LCN1) can cross-react with dog lipocalin Can f 1, which is likely due to their high sequence identity of 61% [33]. Additionally, both Can f 1 and Fel d 7 (cat lipocalin) share 57.7% sequence identity with human lipocalin-1 (encoded by the LCN1 gene), and the presence of sIgE to these allergens could serve as a marker of the severity of atopic dermatitis (AD) [34]. This molecular similarity underlies the observed IgE cross-reactivity between animal and human lipocalins, although the clinical significance of this phenomenon appears limited. Nonetheless, it provides important insight into the broad cross-reactivity seen in individuals sensitized to animal lipocalins and may have implications for disease phenotyping and severity assessment.

### Potential for Cross-Reactivity of Lipocalins

From a physician’s perspective, it is crucial to identify potential cross-reactions and assess their likelihood of occurrence. The recent study by Chruszcz et al. proposed a novel methodology for assessing the likelihood of cross-reactivity within members of protein families, which was computed based on sequence similarities, identities, and reports on clinically relevant cross-reactivities. Researchers introduced the A-RISC index (Allergens’-Relative Identity, Similarity, and Cross-reactivity), with four levels describing the probability of cross-reactions as high (A-RISC ≥ 0.75), medium–high (0.75 > A-RISC ≥ 0.50), medium–low (0.50 > A-RISC ≥ 0.25), and low (A-RISC values < 0.25). Among 10 analyzed allergen families (papain-like cysteine proteases, NCP2 family, serum albumins, pectate lyases, lipocalins, β-expansins and expansin-related, Group V/VI grass pollen allergens, profilins, non-specific lipid transport proteins, Bet v 1 family (PR-10 s)), lipocalins were ranked the lowest in terms of likelihood of cross-reactivity. Considering the wide range of allergenic proteins in the lipocalin group and the variability in amino acid sequences, Chruszcz et al. have established a categorization into seven distinct subgroups, which we summarized and presented in Table 1.

**Table 1:** Summary of potential cross-reactivity risk among lipocalin subgroups based on existing analyses of Chruszcz et al. 2018 [35]

Subgroup	Class	Order	Proteins	Risk of cross-reactivity
1	Insecta	Blattodea	Bla g 4, Per a 4,	Low
		Hemiptera	Tria p 1	
	Arachnida	Sarcoptiformes	Arg r 1	
2	Arachnida	Sarcoptiformes	Aca s 13, Blo t 13, Der f 13, Der p 13, Lep d 13, Tyr p 13	High
3	Mammalia	Carnivora	Can f 1, Fel d 7	High
4	Mammalia	Artiodactyla	Bos d 5, Sus s 5	High
5	Mammalia	Artiodactyla	Bos d 2	Medium–low
		Carnivora	Can f 4	
		Rodentia	Cav p 2, Cav p 3, Mes a 1, Phod s 1	
		Lagomorpha	Ory c 1	
6	Mammalia	Carnivora	Can f 2	Low
		Rodentia	Most similar to Rat n 1	
7	Mammalia	Artiodactyla	Bos gr 1	Medium–high
		Carnivora	Can f 6, Fel d 4,	
		Perissodactyla	Equ c 1	
		Rodentia	Cav p 6, Mus m 1, Rat n	

Aca s, *Acarus siro*; Arg r, *Argas reflexus*; Bla g, *Blattella germanica*; Blo t, *Blomia tropicalis*; Bos d, *Bos domesticus*; Bos gr, *Bos grunniens*; Can f, *Canis familiaris*; Cav p, *Cavia porcellus*; Der f, *Dermatophagoides farinae*; Der p, *Dermatophagoides pteronyssinus*; Equ c, *Equus caballus*; Fel d, *Felis domesticus*; Lep d, *Lepidoglyphus destructor*; Mes a, *Mesocricetus auratus*; Mus m, *Mus musculus*; Ory c, *Oryctolagus cuniculus*; Per a, *Periplaneta americana*; Phod s, *Phodopus sungorus;* Rat n*, Rattus norvegicus*; Tria p, *Triatoma protracta*; Tyr p, *Tyrophagus putrescentiae*

Apart from sequence identity and sequence similarity of allergens, the presence of conformational epitopes may also impact the risk of cross-reactivity, as experimental research suggests [36].

Among the lipocalins from various animals, as of the present moment, 19 proteins have been officially recognized and listed by the World Health Organization and International Union of Immunological Societies (WHO/IUIS) Allergen Nomenclature Sub-Committee [37]. Lipocalins have been identified as allergens in the following animals:

- cat - Fel d 4, Fel d 7;
- dog - Can f 1, Can f 2, Can f 4, Can f 6;
- horse - Equ c 1, Equ c 2;
- cow - Bos d 2, Bos d 5;
- guinea-pig - Cav p 1, Cav p 2, Cav p 3,
- rabbit - Ory c 1; Ory c 2;
- rat - Rat n 1;
- mouse - Mus m 1;
- hamster: Syrian hamster – Mes a 1; Siberian hamster - Phod s 1.

Lipocalins have also been identified in prairie dogs (feces contaminated with urine), chinchillas, and gerbils [38–40]. However, so far, we have limited diagnostic tools (sIgE/SPT) to pinpoint the causative allergens from exotic animals [41]. A complete list of lipocalins is provided in Table 2. To emphasize clinically significant molecules, all allergens listed in Tables 2, 3, and 4 were classified as either major or minor allergens according to their sensitization rates among allergic subjects. Major allergens are characterized as those that are detected by IgE in serum in over 50% of individuals allergic to the respective allergenic sources, while minor allergens are noted in fewer than 50% of patients [11, 42].

**Table 2:** Lipocalins and their distinctive characteristics

Animal	Latin name	Allergen	Clinical relevance		Production organ	Source of allergen	Route of exposure	Comparison of diagnostic tests by the range of allergen tested		
			Major/minor	Sensitization rate among patients				ALEX2 ®	ImmunoCAP™	ImmunoCAP ISAC
Cat	*Felis domesticus*	Fel d 4 [43]	Major allergen	63%	Salivary gland	Saliva, dander, urine	Airway	√	√	√
		Fel d 7 [44]	Minor allergen	38%	Ebner glands on feline’s tongue	Saliva, hair	Airway	√	√	
Dog	*Canis familiaris*	Can f 1 [45]	Major allergen	50–90%	Tongue	Saliva	Airway	√	√	√
		Can f 2 [46]	Minor allergen	22–35%	Tongue	Saliva	Airway	√	√	√
		Can f 4 [47]	Minor allergen	35–49%	Tongue	Dander	Airway	√	√	√
		Can f 6 [48]	Minor allergen	56%	Submaxillary gland	Dander	Airway	√	√	√
Horse	*Equus caballus*	Equ c 1 [49]	Major allergen	76–100%	Sublingual gland	Saliva, dander, urine	Airway	√	√	√
		Equ c 2 [50]	Minor allergen	33–62%	nd	Dander, sweat	Airway			
Cow	*Bos domesticus*	Bos d 2 [51]	Major allergen	90%	Sweat glands	Skin	Airway	√		
		Bos d 5 [52]	Major allergen	90%	Mammary gland	Milk	Food	√	√	√
Guinea-pig	*Cavia porcellus*	Cav p 1 [53]	Major allergen	70%	Harderian gland	Hair, urine	Airway	√		
		Cav p 2 [53]	Major allergen	62%	Harderian gland	Hair	Airway			
		Cav p 3 [54]	Major allergen	45–54%	Submaxillary gland	Hair, saliva	Airway			
		Cav p 6 [55]	Minor allergen	18%	Harderian gland	Hair	Airway			
Rabbit	*Oryctolagus cuniculus*	Ory c 1 [56]	Major allergen	90%	nd	Saliva, hair, urine	Airway	√		
		Ory c 2 [57]	Major allergen	75%	nd	Saliva, hair, urine, dust	Airway	√		
		Ory c 4 [58]	Minor allergen	46%	nd	Hair and dust	Airway			
Rat	*Rattus norvegicus*	Rat n 1 [59, 60]	Major allergen	90%	Liver	Urine, saliva, fur	Airway	*		
Mouse	*Mus musculus*	Mus m 1 [61]	Major allergen	90%	Liver	Urine, saliva, hair	Airway	√	√	√
Siberian hamster	*Phodopus sungorus*	Pho s 1 [62]	Major allergen	90%	Salivary gland	Hair, urine	Injection	√		
Golden hamster /Syrian hamster	*Mesocricetus auratus*	Mes a 1 [63, 64]	Major allergen Male-specific allergen [31]	66–83%	Male-specific submandibular salivary gland protein	Fur	Airway	*		

*nd*, no data; *Rat n 1 and Mes a 1 will be available on ALEX3 ®

### Clinical Relevance of Lipocalins

As of today, eight allergens have been identified in dogs that elicit allergic reactions in humans. Among these allergens, four are classified as lipocalins: Can f 1, Can f 2, Can f 4, and Can f 6. In a study conducted in Western Sweden by Özuygur Ermis, which examined sensitization to various components of dogs (namely Can f 1, Can f 2, Can f 3, Can f 4, Can f 5, and Can f 6 utilizing ImmunoCAP™) among 313 extract-sensitized adults, it was found that lipocalins represented the predominant sensitizing family of allergens [74].

In a study investigating the sensitization profile among children (*n* = 60) with positive skin prick test (SPT) results to dog dander extract and/or IgE to dog dander extract, irrespective of clinical history, the authors examined the sensitization to Can f 1, Can f 2, Can f 3, Can f 4, Can f 5, and Can f 6 utilizing ImmunoCAP™. The data demonstrated that polysensitization to an increasing number of dog allergens was correlated with a positive nasal provocation test (NPT) [75]. Käck et al. in their study showed that polysensitization to lipocalins, as well as sensitization to Can f 4 (OR, 6.80; 95% CI, 1.84–25.2) and Can f 6 (OR, 5.69; 95% CI, 1.59–20.8), was also associated with a positive NPT. Therefore, the authors concluded that sensitization to Can f 4 and Can f 6 may indicate clinically relevant dog allergy. Additionally, a rising number of sensitizing dog allergen components and lipocalins is associated with dog allergy and increased the risk of reporting dog-induced allergic rhinitis or asthma [76].

Moreover, another study observed a correlation between polysensitization and the severity of asthma [11]. Konradsen et al. found that children sensitized to three or more mammalian lipocalin allergens are predisposed to experiencing severe asthma [77]. A study conducted in West Sweden on sensitization to dog allergens (Can f 1, Can f 2, Can f 3, Can f 4, Can f 5, and Can f 6 using ImmunoCAP™) indicated that asthmatic individuals exhibited elevated IgE levels for two lipocalins, Can f 4 and Can f 6, as well as serum albumin, Can f 3, compared to non-asthmatics. Data have also shown that asthma correlated with polysensitization, which was defined as sensitization to ≥ 3 molecules [74].

One of the dog lipocalins is Can f 1, which is a major dog allergen. According to Chruszcz et al., Can f 1 has a high likelihood of cross-reactivity with Fel d 7, partially due to a high percentage sequence identity of 63% [35, 78]. In a comprehensive BAMSE study that evaluated sensitization among 779 randomly selected children from Sweden’s birth cohort at ages 4, 8, and 16, ImmunoCAP™ was utilized to determine sIgE levels to dog allergens (Can f 1; Can f 2; Can f 3; Can f 5; Can f 6) and cat allergens (Fel d 1; Fel d 2; Fel d 3). The findings of the BAMSE study are substantial, indicating that sensitization to Can f 1 is a strong predictor of future dog allergy symptoms [79].

The remaining three dog lipocalins (Can f 2, Can f 4, and Can f 6) are acknowledged as minor allergens. Can f 2 sensitizes 22–35% of dog-allergic patients and demonstrates a low sequence identity of approximately 20–30% with other lipocalins [11]. It is associated with a diagnosis of asthma [5].

Can f 4 is among the most frequently detected allergens in canine fur. However, the extracts utilized for skin prick testing do not accurately reflect this prevalence because they contain inadequate quantities of this allergen [80]. Consequently, the sensitization to Can f 4 in patients with a dog allergy may be underestimated. The final minor allergen, Can f 6 (38–56%), is a highly cross-reactive protein [11, 77]. Research by Nilsson et al. showed that Can f 6 can cross-react with lipocalins derived from cats and horses. This is due to a high degree of amino acid similarity, as Can f 6 shares 80.0% similarity and 67.4% identity with the cat lipocalin Fel d 4. Concerning the horse lipocalin Equ c 1, Can f 6 has a similarity of 72.0% and an identity of 55.1% [81]. Recent findings by Swiontek et al. highlight that guinea pig lipocalin Cav p 6 has a 54% sequence homology with Can f 6 [82]. Can f 6, similar to Can f 4, is associated with clinically relevant dog allergy and associated with rhinitis and asthma among children [5, 74, 76].

The allergy to felines is the most prevalent form of furry animal allergy. Among the eight known cat allergens, only two (Fel d 4 and Fel d 7) are classified within the biochemical family of lipocalins. Nonetheless, these allergens exhibit significant clinical relevance and should be considered in diagnostic algorithms and immunotherapy.

Cat lipocalin, Fel d 4, is the major allergen of felines and is primarily found in cat saliva; it is responsible for sensitizing around 63% of individuals with cat allergies [83]. It can cross-react with proteins such as Can f 6 (sequence identity 67%), Equ c 1 (sequence identity 67%), and Cav p 6 (sequence identity 53%). The second lipocalin, Fel d 7, is a minor cat allergen that has the potential to cross-react with Can f 1 (sequence identity 62%). It is produced by Ebner’s glands on the tongues of cats and is secreted into saliva.

A study conducted by Wisniewski et al. investigated sensitization to different allergens (including cat allergens Fel d 1, Fel d 2, and Fel d 4) in 66 children with active atopic dermatitis (AD). The findings indicated that sensitization to Fel d 4 (titers > 0.3 IU/mL) was associated with wheezing in cat-allergic patients with AD (*p* = 0.04) [84]. In another study examining sensitization to cat allergens (Fel d 1, Fel d 2, Fel d 4, Fel d 7) within the adolescent population (*n* = 266) diagnosed with asthma, the levels of IgE to Fel d 4 (and Fel d 2–serum albumin) were found to be independently correlated with type-2 biomarkers as well as total IgE levels in young individuals affected by asthma (*p* = 0.009) [85]. According to a recent study testing cat allergens (Fel d 1, Fel d 2, Fel d 3, Fel d 4, Fel d 6, Fel d 7, and Fel d 8.) on a sensitized population of 2 groups: symptomatic patients (*n* = 37) and asymptomatic individuals (*n* = 20), results have shown that Fel d 4, Fel d 7, and Fel d 1 induced the maximum basophil activation at a low dose in symptomatic children. These findings suggest that Fel d 1, Fel d 4, and Fel d 7 should be considered in the diagnosis and qualification of specific allergen immunotherapy in patients allergic to cats [86].

In the context of lipocalins, an interesting study by Hemmer et al. addresses molecular sensitization patterns in adults and children with suspected allergic rhinitis or asthma and positive skin test results in cats, dogs, and/or horses. IgE analysis performed using ImmunoCAP™ against a broad profile of cat, dog, and horse proteins (Fel d 1, 2, 4, 7, Can f 1, 2, 3, 4, 5, 6, and Equ c 1, respectively) showed that double sensitization to cats and dogs (25.9%), cats and horses (5.4%), and polysensitization to cat, horse, and dog (20.7%) is linked with an increased prevalence of the cross-reactive lipocalins Fel d 4/Can f 6/Equ c 1 and Fel d 7/Can f 1. An additional conclusion of this study is that primary sensitization is most often linked to the allergen that causes the highest IgE levels. This discovery enables patients with polysensitization to multiple animal allergens to identify the primary sensitizer by comparing sIgE levels [87].

So far, five allergens originating from horses have been identified. Among them, only two are lipocalins: Equ c 1 and Equ c 2. The first one, Equ c 1, is a major allergen that sensitizes 76–100% of patients allergic to horses. In a study by Konradsen et al. on a Swedish population of children afflicted with severe (*n* = 37) and controlled asthma (*n* = 28), the researchers employed molecular diagnostics to assess the sensitization profiles of asthmatic patients (*n* = 65) with FAA; the findings indicated elevated levels of sIgE to Equ c 1 among individuals experiencing severe asthma (*p* = 0.017). Moreover, an asthma control test demonstrated that increased antibodies against Equ c 1 were associated with diminished asthma control in children (*p* = 0.036) [77]. Additionally, another study investigating the sensitization profile among the West Sweden adult population (*n* = 1872) suggested that sensitization to Equ c 1 constitutes a risk factor for asthma and allergic rhinitis, and it can serve as a marker of asthma severity [88].

### Occupational Allergy

#### Laboratory Animal Allergy (LAA)

When considering the topic of lipocalins, it is crucial to discuss the issue of allergy to small mammals, as this is an underrepresented topic in current guidelines despite the significant risk it poses to laboratory workers, including scientists and technicians. Epidemiological data reveal that 10–30% of laboratory staff experience allergies to rats or mice. In a recent study, 30% of laboratory workers reported symptoms of allergy during exposure to mice, while 13.7% reported symptoms following contact with rats. In both cases, the most common symptom was allergic rhinitis (54.7% and 62.1%, respectively) [89]. In a study by Straumfors et al., researchers scrutinized the level of exposure to Mus m 1 and Rat n 1 in laboratories with animals. Among laboratory workers, animal technicians were most exposed to these allergens. The peak exposure to allergens was recorded during cage emptying and using the cage washroom. In comparison, the lowest exposure was determined during experiments performed on animals in the laboratory or operating room. The study revealed that individually ventilated cages decreased exposure to Mus m 1 and Rat n 1. In contrast, open shelves and sliding doors primarily raised the Rat n 1 exposure [90].

To date, only two singular rat and mouse allergens have been identified: Rat n 1 (*Rattus norvegicus*) and Mus m 1 (*Mus musculus*). They both belong to a group of lipocalin proteins. The primary route of exposure to Rat n 1 and Mus m 1 occurs through inhalation [86, 87]. In the case of these rodents, molecules are produced in the liver and secreted in the urine. Studies indicate that males produce higher levels of Mus m 1 than females, as Mus m 1 is hormonally dependent and androgens stimulate its release. Rat n 1 shares 47% identity with Equ c 1, 52% Can f 6, 55% Fel d 4, and 64% Mus m 1 [11]. In the study by Jeal et al., RAST inhibition was performed, and the findings have shown that dual sensitization to Mus m 1 and Rat n 1 is due to cross-reactivity rather than atopy. This conclusion holds significant relevance [91].

Guinea pigs can also serve as a source of LAA. Among medical researchers engaged in work involving guinea pigs at the Kochi Medical University, 9.7% of individuals reported the presence of allergy symptoms associated with these animals [92]. Among the five allergens identified in guinea pigs, four are classified as lipocalins: Cav p 1, Cav p 2, Cav p 3, and Cav p 6. Major allergens are Cav p 1, Cav p 2, and Cav p 3, with sensitization rates of 70–83%, 62–65%, and 45–54%, respectively [37, 79]. All three allergens individually are markers of genuine sensitization to guinea pigs [82]. Nonetheless, Cav p 2 and Cav p 3 are species-specific, implying their enhanced diagnostic sensitivity. Cav p 2 is produced in the harderian gland, whereas Cav p 3 is produced in the submaxillary gland [41]. Cav p 6 is a minor allergen that may cross-react with Fel d 4, Can f 6, and Equ c 1 due to its high sequence identity [21]. According to Swiontek et al., using these lipocalins—Cav p 1, Cav p 2, Cav p 3, and Cav p 6—enables the identification of up to 90% of patients with an allergy to guinea pig. Furthermore, the authors suggest that the initial step in diagnosing guinea pig allergy should involve a panel of three allergens: Cav p 1, Cav p 2, and Cav p 3. To clearly distinguish between patients who have a genuine allergy to guinea pigs and those with cross-reactions to feline or canine allergens, it is preferable to measure sIgE levels to these three specific allergens rather than to guinea pig extracts. This approach mitigates the risk of obtaining false positive results due to cross-reaction between serum albumins Cav p 4 and Fel d 2 or Can f 3 or by cross-reaction between lipocalins Cav p 6 and Fel d 4 or Can f 6, or Equ c 1 [82]. So far, the concentration of IgE against Cav p 1 can only be measured using the ALEX2 **®** test.

Concerning the rabbit, these animals represent a frequent source of LAA. Among medical researchers at Kochi Medical University working with rabbits, 16.3% of individuals reported experiencing allergy symptoms associated with contact with these animals [92]. In rabbits, 4 allergens have been isolated, 3 of which have the biochemical structure of lipocalins: Ory c 1, Ory c 2, and Ory c 4, and they are found in saliva, urine, and fur. Ory c 1 and Ory c 2 are major rabbit allergens, sensitizing, respectively, in 100% and 71% of rabbit-allergic patients. Ory c 4 is categorized as a minor allergen; the prevalence of sensitization among individuals allergic to rabbits is 46% [93]. Ory c 4 has high sequence similarity to Can f 6 (58%), Fel 4 (63%), and Equ c 1 (52%), which suggests a high probability of cross-reaction [11].

**Fig. 1:**
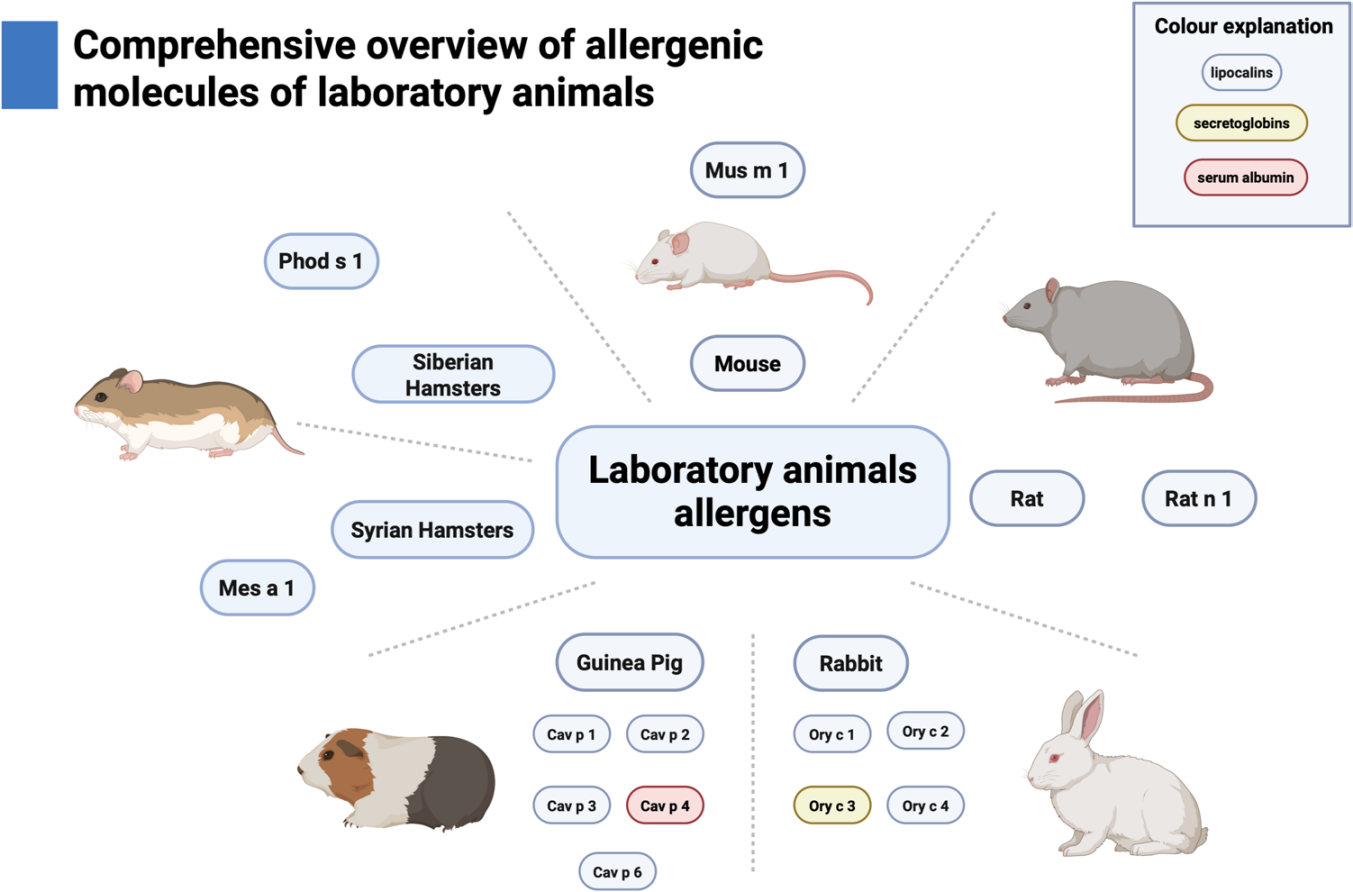
Laboratory animals and their allergenic molecules. Created with BioRender.com [101]

The final rodent to consider regarding occupational hazards is the hamster, a common laboratory and pet animal. In a 2023 literature review on anaphylaxis following animal bites, seven cases were documented after bites from hamsters [94]. In the WHO/ISUS database, there are two allergens associated with two species of the *Cricetidae* family [95, 96], which have lipocalins: Mes a 1 and Phod s 1. The first, Mes a 1, is the only allergen of Syrian/Golden hamsters. The highest concentration of Mes a 1 is found in the saliva and urine of males, but it is also present in lower concentrations in the tears of females [97, 98]. Mes a 1 Syrian hamster (*Mesocricetus auratus*) shows a 40% sequence identity with Phod s 1 Siberian hamster (*Phodopus sungorus*), which belongs to one of the smallest hamster species naturally found in Russia and Kazakhstan. There were three cases of anaphylaxis following bites from *Phodopus sungorus*, including two among children [98–100]. A visual summary of laboratory animal allergens is presented in Fig. 1.

#### Cow Epithelium Allergy (CEA)

Another occupational hazard encountered by farmers pertains to cattle, specifically cow epithelium allergy [102]. In a retrospective observational study conducted in Germany that focused on allergy among farmers with a suspected occupational cattle allergy (*n* = 513), researchers discovered that 24.8% (*n* = 127) of the farmers experienced asthma related to exposure to cattle, 11.7% (*n* = 60) reported rhinitis, and 60% (*n* = 308) exhibited symptoms of both asthma and rhinitis [103]. In a recent study investigating the clinical manifestations of allergic reactions among CEA patients, data revealed that all individuals exhibited rhinoconjunctivitis symptoms, 38% had asthma, and 29% experienced urticaria. The predominant sensitization pattern identified in the study revealed that 41% of patients exhibited polysensitization to cow epithelia, house dust mites, and/or pollens. Additionally, 35% of patients diagnosed with CEA were monosensitized to cow epithelia [102]**.**

For the farmers, the workplace constitutes the most hazardous environment for the exacerbation of allergic diseases, given the significantly elevated concentrations of Bos d 2, which exhibit a median value of 20,400 μg/g in barns and stables. Research shows, however, that farmers are also exposed to bovine allergens outside of their workplace environments. Dust samples collected from their living rooms and mattresses have surpassed the threshold of 20–50 μg/g of Bos d 2, which is necessary for sensitization [104]. It is noteworthy that there may be a novel major allergen in addition to Bos d 2. Guzman et al. identified that 70% of patients reacted to a 25-kDa protein found in urine extracts from cattle, specifically cows and bulls. This identification was based on mass spectrometry and a search of protein databases, indicating correspondence to the lipocalin of *Bos taurus* [102, 105]*.*

Among the allergenic proteins of cows are two lipocalins. Bos d 2 is identified as a major cow allergen present in sweat glands, dander, and urine. In contrast to Bos d 2, which is an inhalant allergen, another major bovine allergen, Bos d 5—beta-lactoglobulin, is a food allergen found in whey, the liquid part of milk [52]. Additionally, from a clinical perspective, it is crucial to distinguish CEA from milk allergy, beef allergy, and alpha-gal syndrome, as distinct allergens provoke these conditions and exhibit slightly varied symptoms. Alpha-gal (glycan galactose-alpha-1,3-galactose) is one type of carbohydrate residue. Cross-reactive carbohydrate determinants are sugar residues of diverse structures present in various allergens and possess the capacity to elicit an immune response in humans [106]. However, they do not induce clinical manifestations, except for alpha-gal syndrome. Consequently, to differentiate a genuine beef allergy from an allergy to alpha-gal, it is imperative to assess IgE to Bos d 5 and alpha-gal [107]. An allergy to alpha-gal is a type of delayed IgE hypersensitivity to non-primate mammalian meat (e.g., beef, pork, or lamb) and other products containing alpha-gal, such as certain drugs like cetuximab. Symptoms occur 5–6 h after ingestion of a product containing alpha-gal. Contemporary research suggests that primary immunization occurs by tick bite [108].

It is worth mentioning that there is a group of minor allergens identified in felines that have an immunoglobulin structure—Fel d 5, which has a structure of immunoglobulin A, while Fel d 6 is an immunoglobulin M [109, 110]. Both allergens have an IgE epitope, alpha-Gal [111, 112]. In light of this information, an allergy to furry animals, particularly cats and dogs, can be problematic for patients with AGS, as they may receive false-positive results for cat and dog extracts used in skin testing due to the presence of alpha-gal in these products. Therefore, authors suggest that physicians should consider testing patients with AGS for components Fel d 1, Fel d 4, Can f 1, and Can f 4 to confirm the diagnosis of allergy to a specific animal [113, 114]. However, there are no studies examining whether individuals sensitized to Fel d 5 or Fel d 6 are at risk of developing AGS. Further research is needed on this topic. Although these cat allergens contain alpha-gal, it is unclear whether there is a risk of cross-reactivity with mammalian meat and other products that contain alpha-gal.

#### Key Points

- Lipocalins represented the predominant sensitizing family of allergens from furry animals.
- Can f 2 and Can f 4 are markers of primary sensitization to dogs.
- Cav p 2 and Cav p 3 are markers of genuine guinea pig allergy, which are species-specific.
- Polysensitization to three or more lipocalins is a marker of severe asthma.
- Fel d 4 is related to the diagnosis of asthma.
- Fel d 1, Fel d 4, and Fel d 7 should be considered in the diagnosis and immunotherapy of patients allergic to cats.
- Due to the high potential for lipocalins to cross-react with other species, the level of sIgE should be considered an indicator of the primary source of sensitization. In the case of cross-reactive lipocalins (Fel d 4, Can f 6, and Cav p 6), we should consider the allergen with the highest sIgE level to determine the primary sensitizer.
- Equ c 1 is a marker of severe asthma and poor asthma control.
- Higher levels of specific sIgE to animals’ lipocalins, such as those of cats, dogs, and horses, are markers of asthma severity and genuine allergy.
- In addition to rats and mice, guinea pigs, hamsters, and rabbits are laboratory animals that can constitute occupational hazards.
- Cow epithelia allergy is common among cattle farmers. Very often, symptoms include asthma or rhinitis following exposure to Bos d 2.

## Serum Albumins

Serum albumins (SA) are the second most common family of allergenic proteins in furry animals. They are one of the most significant allergens for single protein chains (approx. 580 amino acids). They have a primary α-helical structure and size of 70 kDa [115]. They are primarily found in mammals; therefore, they are not as diversified a family as lipocalins. Albumins, which are present in animal blood serum, milk, and meat, serve various functions. They can adapt their conformation, effectively binding and transporting multiple molecules such as metabolites, nutrients, and drugs [115]. They also contribute to maintaining oncotic pressure and balancing blood pH [116].

It is recognized that proteins with sequence identity to human homologs above 62% rarely cause sensitization—the serum albumin group is an exception. Despite having high sequence identity (72–82%) to human serum albumins, they can induce IgE-dependent hypersensitivity [115, 117]. Currently, the WHO/IUIS database describes seven serum albumins in the animal kingdom; among them, six are identified in mammals (dog—Can f 3, cat—Fel d 2, cow—Bos d 6, horse—Equ c 3, guinea pig—Cav p 4, domestic pig—Sus s 1) and one in birds: chickens (Gal d 5) [20]. This group of allergens is categorized as inhalants, except for Bos d 6, Sus s 1, and Gal d 5 molecules, which are considered food allergens [118, 119].

### Clinical Relevance of Serum Albumins and Their Cross-Reactivity

Cross-reactivity between various animals may be caused by the presence of sIgE in serum albumins [117]. As per the A-RISC Index developed by Chruszcz et al., serum albumins have a high likelihood of provoking cross-reactions. They were ranked second after profilins in terms of cross-reactivity among ten allergenic protein families [35].

One example of such albumin cross-reactivity is cat-pork syndrome, described in 1994 by Drouet et al. [120]. This syndrome manifests as a broad spectrum of allergic reactions (from mild urticaria to severe anaphylaxis) after consuming pork meat in subjects that are sensitized to cat serum albumin. This can be explained by high sequence similarity (79%) between two serum albumins: Fel d 2 and Sus s 1 [120, 121]. This syndrome affects 1–3% of people with Fel d 2 allergies [121]. The onset of symptoms typically occurs within 30 to 45 min after consuming pork [122]. Symptoms of allergy may also occur after consumption of beef or broiled beef intestines [122, 123]. It is worth noting that patients tend to experience the most severe reactions when consuming raw or undercooked meat, such as in sausages or ham. Beef and pork are usually well tolerated after subjecting meat to high temperatures because albumins are thermolabile. Therefore, fresh meat or dried and smoked pork are more common triggers than well-cooked meat [124]. One of the methods that significantly reduces the allergenicity of Sus s 1 is the autoclave treatment of pork serum albumin at 121 °C for 30 min [125].

In the literature, anaphylaxis has been described as occurring after the use of surgical tissue adhesive containing bovine serum albumin (BSA), specifically due to the cat-pork syndrome, which is caused by the patient’s sensitization to Fel d 1 and Fel d 2. Given that Fel d 2 may exhibit cross-reactivity with mammalian serum albumins, performing IgE diagnostics for Fel d 2 in individuals allergic to cats can help determine the suitability of using bovine and porcine surgical products [122, 126].

It is worth highlighting that the BSA is widely utilized in various applications, including culture media for artificial insemination and vaccine production [127, 128]. Case reports have been reported of women allergic to BSA experiencing anaphylaxis after intrauterine insemination (IUI) or in vitro fertilization. In a case report by Orta et al., a 33-year-old female experienced an anaphylactic reaction after a second intrauterine insemination (using Upgraded B2 INRA medium; Laboratories CCD, Paris, France). She had a history of pollen allergy and asthma attributed to cat epithelium. A cat was present at her house. Diagnosing the cause of anaphylaxis, positive results from SPT and sIgE have been obtained from semen culture, albumin extracts, and sera from pig, horse, cat, dog, and bovine origin. Negative sIgE results were found for seminal fluid, latex, ethylene dioxide, formaldehyde, and antibiotics used in the medium, including penicillin G and V. The authors suggest that cat albumin was the most likely sensitizing agent that cross-reacted with BSA. The authors recommended performing SPT tests with a medium for patients undergoing artificial insemination [129]. In the other case report by Pagan, a 30-year-old woman also experienced an anaphylactic reaction after artificial insemination with spermatozoid in a medium containing BSA (Upgraded B2 INRA medium). She also had a history of respiratory allergy (asthma/rhinitis) to house dust mites, epithelia, and grass pollen. In diagnosing the cause of anaphylaxis, positive SPT results were found in mites, grasses, Olea pollens, and epithelia from cats, dogs, horses, and rabbits, as well as an upgraded B2 INRA medium. The ImmunoCAP™ test showed positive sIgE results for cat, dog, and rabbit epithelium, as well as cat, dog, pig, and bovine serum albumins, horse serum proteins, and Fel d 1[127]. Both studies by Ort and Pegan suggest the utilization of semen cultures without BSA for artificial insemination in the case of cat-allergic patients. However, these studies were conducted at a time when molecular diagnostics had not yet been sufficiently developed [127, 129]. In a 2021 review on the state-of-the-art biomarkers for anaphylaxis in obstetrics, the authors suggested that Fel d 2 and Bos d 6 could serve as biomarkers for potential anaphylaxis in cat-allergic patients undergoing intrauterine insemination or in vitro fertilization procedures [130].

Another example that exemplifies the considerable cross-reactivity of serum albumins is a case study conducted by Morisset et al., which describes a patient with a dog allergy who experienced anaphylaxis after consuming horse meat. The primary reason was sensitization to dog serum albumin Can f 3, which cross-reacted with horse serum albumin—Equ c 3 [131]. Among other examples of cross-reactivity, there is an interesting case of anaphylaxis in a 10-year-old boy following consumption of raw horse meat. The patient had a history of severe AD. Additionally, he lived in an environment where a cat was present, and contact with this animal resulted in AD and asthma exacerbations. To diagnose the reason for anaphylaxis, both ImmunoCAP™ and ImmunoCAP ISAC tests were performed, revealing positive results for Fel d 2, Equ c 3, Sus s 1, Bos d 6, and Can f 1, while negative to α-gal. The authors propose that the patient was initially sensitized to cats and subsequently experienced anaphylaxis due to cross-reaction with Fel d 2 and Equ c 3. Furthermore, the authors suggest that the primary sensitization to dogs is unlikely to be true since the patient had no contact with dogs. The positive result for Can f 1 is probably caused by its cross-reactivity with Fel d 7, an allergen that is not present on ImmunoCAP ISAC[132].

**Fig. 2:**
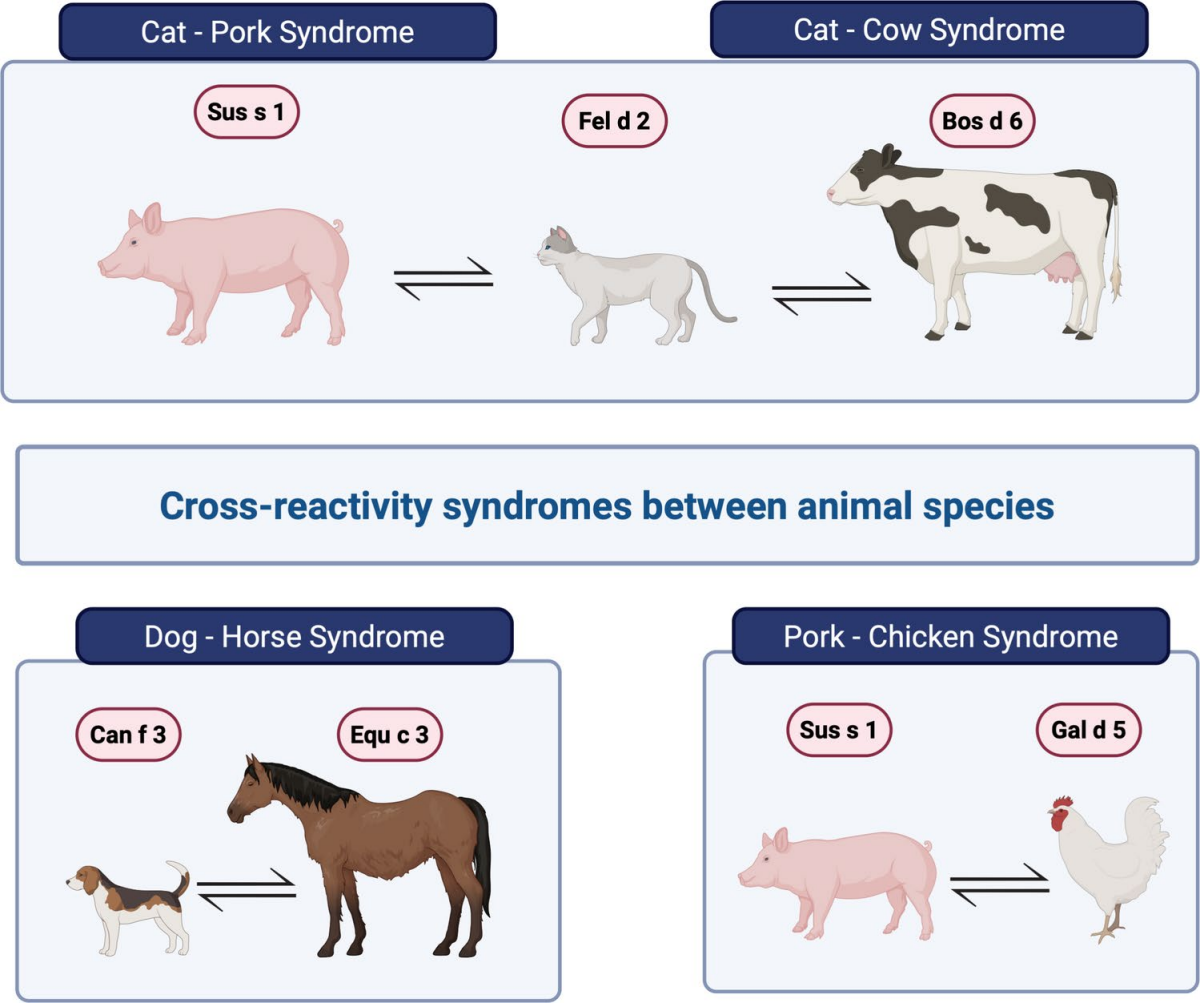
Examples of cross-reactive syndromes between animals due to serum albumins. Created with BioRender.com [134]

**Fig. 3:**
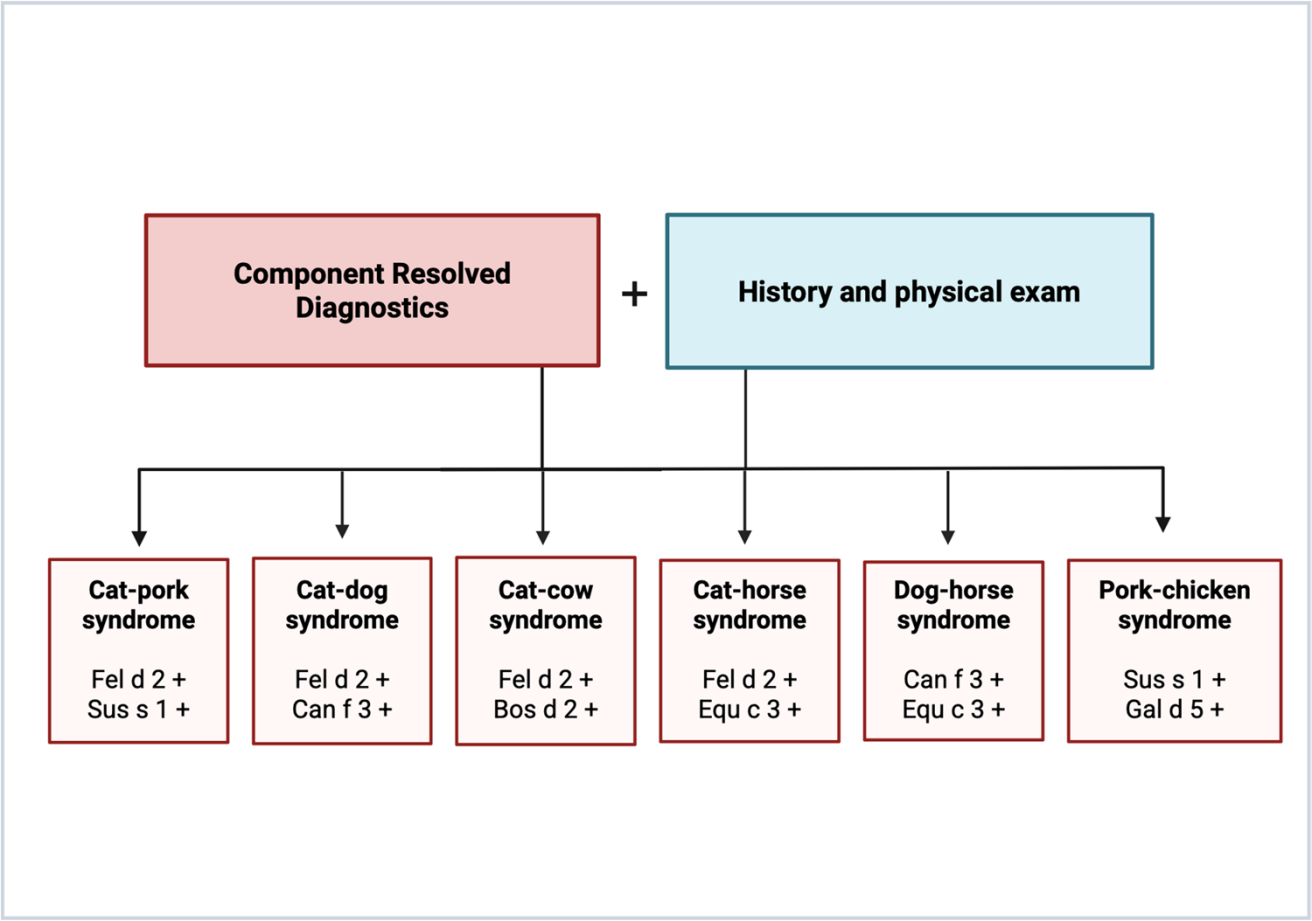
The application of molecular diagnostics in the identification of clinical syndromes associated with cross-reactivity of serum albumin. Created with BioRender.com [135]

 Another research group has also documented a notable case highlighting the high cross-reactive potential of serum albumin. Hilger et al. describe a female employee at a poultry factory who experienced a sequential inhalant allergy to pork, subsequently developing food allergies to both pork and, 3 years later, chicken. The authors suggest that the observed cross-reactivity between Sus s 1 and Gal d 5 may be linked to prior sensitization to cat serum albumin [133]. A summary of SA can be found in Table 3, and examples of cross-reactions of SA are displayed in Fig. 2. The presentation of the application of component-resolved diagnostics for clinical syndromes occurring due to cross-reactivity of SA is shown in Fig. 3.

**Table 3:** Serum albumins and their distinctive characteristics

Animal	Latin name	Allergen	Clinical relevance		Production organ	Source of allergen	Route of exposure	Comparison of diagnostic tests by the range of allergen tested		
			Major/minor allergen	Sensitization rate among patients				Alex2®	ImmunoCAP™	ImmunoCAP ISAC
Cat	*Felis domesticus*	Fel d 2 [65]	Minor allergen	14–23%	Liver	Saliva	Airway	√	√	√
Dog	*Canis familiaris*	Can f 3[66]	Minor allergen [11]	25–60%	Liver	Dander, hair, saliva	Airway	√	√	√
Horse	*Equus caballus*	Equ c 3 [67]	Minor allergen	50%	Liver	Dander	Airway	√		√
Guinea pig	*Cavia porcellus*	Cav p 4[68]	Minor allergen	41%	Liver	nd	Airway			
Domestic cow	*Bos domesticus*	Bos d 6[69]	Major allergen	90%	Liver	Meat, milk, and dander	Food	√	√	√
Domestic pig	*Sus scrofa*	Sus s 1[70]	Minor allergen	Single cases of cat allergic patients	Liver	Dander, meat, milk, and saliva	Food	√		
Chicken	*Gallus domesticus*	Gad d 5[71]	Major allergen	83–100%	Liver	Egg yolk	Food	√	√ (research only)	√

*nd, *no data

#### Key Points

- Serum albumins are thermolabile.
- SA furry animals, despite their high similarity to human SA, can induce hypersensitivity reactions.
- SA are highly cross-reactive and can cause cat-pork, dog-horse, and egg-bird syndromes.
- Fel d 2 and Bos d 6 could serve as biomarkers for potential anaphylaxis in cat-allergic patients undergoing artificial insemination.
- Sensitization to Can f 3 is associated with risk of asthma and allergic rhinitis [77, 88].
- Sensitization to Fel d 2 is associated with symptoms of asthma and moderate to severe allergic rhinitis [5].

## Secretoglobins

So far, only two allergens from this family have been described by WHO/IUIS, one in cats: Fel d 1 and another one in rabbits: Ory c 3 [72, 73]. The secretoglobin Fel d 1 has a molecular weight of 38 kDa. It is a tetramer composed of two non-covalently linked disulfide-linked heterodimers of chains 1 and 2. Ory c 3 is a glycosylated heterodimer of 18 to 19 kDa composed of 2 polypeptide chains, CL2 and AL [136–138]. Ory c 3 exhibits significant structural similarity to Fel d 1 but low identity of 24% [138]. Furthermore, there is no evidence of clinical cross-reactivity between these two allergens [137]. Both allergens are significant allergens. Fel d 1 sensitizes 90% of patients with cat allergy, while Ory c 3 sensitizes 77% of those with rabbit allergy [137, 139].

In the study exploring sensitization profiles to animal molecules using the ImmunoCAP ISAC microarray (cat—Fel d 1, Fel d 2, Fel d 4, dog—Can f 1, Can f 2, Can f 3, Can f 5, horse—Equ c 1, Equ c 3, mouse—Mus m 1, bovine—Bos d 6) among the central European population, the highest sensitization rate was observed for Fel d 1 (31.8%) [140]. Fel d 1, also known as uteroglobin, is synthesized in significant amounts within the salivary, anal, and sebaceous glands of felines, subsequently being transmitted to their fur and skin during grooming activities [141]. The biological role of Fel d 1 remains a mystery; however, studies suggest that it may be involved in chemical communication between cats [142]. All felines generate the protein Fel d 1; however, the quantity of this allergen varies, primarily due to the hormonal status of the individual animal. Male cats typically produce higher amounts than females or neutered males [143, 144]. Furthermore, the amount of this allergen varies by the anatomical site of the feline, with the facial area containing a greater volume of Fel d 1 than the chest region [145].

Fel d 1 is an airborne protein that sticks to small particles. Sixty percent of airborne Fel d 1 is transported by small particles, of which 75% exceed five microns in diameter and 25% measure less than 2.5 microns [146]. As a result, this allergen is widely present in the environment, as observed in a study from the USA. It was found in sofas, carpets, and beds in homes, regardless of whether cats were present in these houses, with occurrences in 99.9% of American households [147].

### Clinical Relevance

Regarding the clinical relevance of sensitization to Fel d 1, it is recognized as a primary marker of cat allergy [139]. Fel d 1 is acknowledged as a marker of authentic feline allergy and should be regarded as one of the paramount allergens considered in the diagnostic algorithms for cat allergy [86, 148, 149].

Sensitization to this allergen does not correlate significantly with feline ownership and is prevalent among individuals who do not own cats [87]. Data shows that the level of sIgE against Fel d 1 is strongly linked to clinical markers of asthma severity [150]. A prospective cohort study by Perzanowski et al. examined 963 individuals aged 19 in Northern Sweden, investigating various cat allergens (Fel d 1, 2, 3, 4, 5) and dog allergens (Can f 1, 2, 3, 5) and their association with asthma symptoms, diagnosis, and treatment; they found that IgE to Fel d 1 is clinically related to current asthma. Additionally, elevated levels of sIgE to cat and dog allergens are linked to the diagnosis, severity, and chronicity of asthma [151]. Individuals sensitized to Fel d 1 can also be sensitized to other Felidae species, including the puma, Siberian tiger, lion, jaguar, and snow leopard, since these species produce substantial amounts of Fel d 1-like protein [152]. There has even been a case of mild anaphylaxis in a cat-allergic 8-year-old boy after indirect contact with a lion during a circus performance [153]. A summary of secretoglobins is presented in Table 4 below.

**Table 4:** Secretoglobins and their distinctive characteristics

Animal	Latin name	Allergen	Clinical relevance		Production organ	Source of allergen	Route of exposure	Comparison of diagnostic tests by the range of allergen tested		
			Major/minorallergen	Sensitization rate amongpatients				Alex2®	ImmunoCAP™	ImmunoCAP ISAC
Cat	*Felis domesticus*	Fel d 1 [72]	Major allergen	90%	Sebaceous, anal, and salivary glands	Fur and epidermis	Airway	√	√	√
Rabbit	*Oryctolagus cuniculus*	Ory c 3 [73]	Major allergen	77%	nd	Fur	Airway	√		

*Nd*, no data

#### Key Points

- Fel d 1 is a marker of genuine cat allergy.
- The level of Fel d 1-specific IgE is associated with asthma severity in cat-allergic patients.
- Patients sensitized to Fel d 1 can develop clinical symptoms when encountering other Felidae animals, such as pumas, Siberian tigers, lions, jaguars, and snow leopards.
- Ory c 3 is a major allergen in rabbits.

## Other Allergens

Patients with allergies to furry animals can be sensitized to multiple allergens, which are not limited to the three groups described above. In addition to lipocalins, serum albumins, and secretoglobins, rarer allergens from furry animals have been identified. To date, the WHO/ISUS has listed allergenic proteins from the following families: cystatin A, immunoglobulin, latherin-like protein, latherin, lysozyme, caseins, myosin light chain, alpha-lactalbumin, and beta-lactoglobulin.

One allergen among the aforementioned requires distinct attention: Can f 5, major dog allergen [154]. Can f 5 is known as kallikrein or arginine esterase. It is produced in the prostate of dogs, and androgens regulate its release. It is present in urine and dander only in male dogs. It has not been found in female canines. The prevalence of sensitization to Can f 5 among dog-allergic patients equals 76%. The conjunctival allergen provocation tests have proven that monosensitization to Can f 5 in individuals with an allergy to dogs suggests tolerance to females of this species [108, 155].

Additionally, the amino acid sequence of Can f 5 shares a similarity of 55–60% [156] with prostate-specific antigen (PSA) found in human seminal plasma (HSP), and this allergen could cross-react with PSA [157, 158]. This may be potentially an explanation for the anaphylaxis reactions among women who are allergic to HSP. Several hypotheses suggest that Can f 5 may be linked to infertility issues; further research is required. To date, we possess clinical case reports [158, 159] and several original research studies [157, 160]; however, their outcomes have not provided statistically significant conclusions.

## Diagnostics

In 2022, the European Academy of Allergy and Immunology published guidelines in Molecular Guide 2.0 on the treatment of allergies to cats, dogs, and horses. This methodology involves taking a clinical history, including questions about contact with animals or the presence of pets at home. Further steps involve conducting SPT or measuring specific IgE levels and, in some instances, using nasal provocation tests to confirm the diagnosis.

SPT is a widely utilized method that involves applying an extract to the skin while observing the reaction. It serves as the initial diagnostic step for allergic diseases and is both cost-effective and reliable. However, caution is necessary when addressing dog allergies, as the concentrations of major dog allergens in commercially available extracts vary among manufacturers. The use of the extract in allergy diagnosis may lead to the underdiagnosis of patients with dog allergies [11].

Another step in diagnosis involves measuring IgE levels in patients’ serum for specific allergens. This method utilizes enzyme-linked immunosorbent assays (ELISA). Depending on the individual patient’s profile, including the range and variety of symptoms experienced, physicians select either a single allergen or a set of allergens for the diagnostic process [161]. Assessing antibodies for specific IgE against cat, dog, or horse allergens is crucial for identifying the source of primary sensitization. Current guidelines do not recommend measuring the total IgE concentration in a patient’s blood, as it does not contribute to diagnosis [11]. Among component-resolved diagnostic tests, we distinguish multiplex tests—ImmunoCAP ISAC, ALEX2**®**, (and the newly announced version of the ALEX3 **®**)—and a singleplex test, ImmunoCAP**™**. Summary of information regarding these diagnostic methods is present in Table 5.

**Table 5:** Comparison of component-resolved diagnostic test

Feature	ImmunoCAP™	ImmunoCAP ISAC	ALEX2®	ALEX3®
Type of method	Singleplex	Multiplex	Multiplex	Multiplex
Allergen panel	Many individual allergens and extracts, each tested separately	112 allergen molecules	178 allergen molecules, 117 extract	218 allergen molecules, 82 extracts
Unit	Kilo units of allergen-specific IgE per liter kUₐ/L	ISAC Standardized Units for specific IgE (ISU-E)	Kilo units of allergen-specific IgE per liter kUₐ/L	Kilo units of allergen-specific IgE per liter kUₐ/L
Strengths	High precision	Simultaneous analysis of many molecules, small amount of serum needed to determine a large number of allergens	Wide allergen panel with both extracts and allergen molecules, CCD blocking, small amount of serum needed to determine a large number of allergens, measurement of total IgE	Broad allergen panel with both extracts and allergen molecules, α-Gal detection, CCD blocking, small amount of serum needed to determine a large number of allergens, measurement of total IgE, expanded number of allergen molecules in comparison to the ALEX2 **®** test
Limitations	Each allergen tested separately, method is time-cosuming and costly for multiple allergens, application of encapsulated cellulose sponge as an allergen carrier that contains sufficient levels of CCD to result in false-positive test results, up to 2 kUA/L, with nonglycosylated recombinant allergens in patients with high levels of anti-CCD IgE antibodies	No extracts, limited number of allergens	Lack of α-Gal allergen	The test is not available for routine application as of July 8, 2025
Citations	[162, 163]	[164, 165]	[166]	[166]

*CCD*, cross-reactive carbohydrate determinants

The final method, NPT, is optional for patients with animal allergies. The nasal provocation test is recommended for complex cases, such as patients allergic to multiple dog molecules. However, in cases of cat allergy, provocation tests are generally unnecessary, as diagnosis using skin tests and sIgE is typically sufficient. Nevertheless, it can be a helpful method when discrepancies exist between SPT and sIgE results or in cases of polysensitization [11].

Diagnostic guidelines have been established for commonly encountered animals, including cats, dogs, and horses. However, there is still a gap in guidelines for allergies related to less popular animal species. As noted in the review by Rosada et al., which explores the diagnostic options for animal allergies, new guidelines are necessary for less prevalent animals [12].

## The Treatment Options

### Current Treatment Options

Currently, the fundamental treatment regimen for furry animal allergies exhibits no substantial variance from that of other forms of allergies. It includes three basic principles: eliminating the causative allergen source, specific immunotherapy, and pharmacological treatment [13].

The most straightforward solution may appear to be removing the animal from the household, but it is often difficult to accomplish. Scientists also propose various pharmacological interventions. The term “pharmacological treatment” is broad and encompasses various classes of drugs, including first-generation and second-generation antihistamines, glucocorticosteroids, anti-leukotriene agents, β2-mimetic agents, anticholinergic agents, immunosuppressive agents, biological agents (such as omalizumab, a humanized monoclonal antibody against IgE), methylxanthines, chromones, and magnesium sulfate [13]. Among the mentioned medications, nowadays, one particular group is significantly transforming the field of allergology: biologic drugs. These treatments are especially effective for severe asthma and atopic dermatitis (AD), as well as other associated conditions. Additionally, we attempted to uncover trustworthy data about the effectiveness of biological drugs in allergic diseases, indeed, caused by furry animal allergens; however, we only found one study regarding omalizumab. Results from a double-blind, placebo-controlled, 28-week pivotal U.S. registration trial have demonstrated that omalizumab is effective in cat-allergic patients (sensitized to Fel d 1) with moderate-to-severe asthma (*n* = 811). Compared to placebo, omalizumab reduced the mean number of asthma exacerbations demanding use of systemic steroids decreased from 1.3 to 0.6 (RR = 0.50, *p* = 0.001), significantly decreased asthma symptom scores (least square means difference = –0.57 [95% CI: –0.77, –0.37]; *p* = 0.001), reduced rescue medication use by 0.75 puffs a day [95% CI: –1.04, –0.46]; *p* = 0.001), and improved forced expiratory volume in one second (FEV₁) by an average of 100.84 mL [95% CI: 51.86, 149.81]; *p* = 0.001). Therefore, for doctors seeking biological treatment options for moderate-to-severe asthma patients who are allergic to cats and sensitized to Fel d 1, they should consider the use of omalizumab. Additionally, this underscores a need for more studies regarding biologic drugs in furry animals with allergic conditions [167]

Allergen-specific immunotherapy (AIT) is a therapeutic approach that aims to desensitize patients suffering from IgE-mediated allergies. We can primarily distinguish between subcutaneous immunotherapy (SCIT) and oral or sublingual immunotherapy (SLIT), as well as novel options such as intralymphatic immunotherapy [168]. Allergies to various animals, including cats, dogs, horses, rabbits, hamsters, guinea pigs, sheep wool, goats, and parrot feathers, can be treated using both SCIT and SLIT. However, for cow hair and parrot feather allergens, only SCIT is available [13]. Furthermore, available options for treating allergies to rodents remain quite limited [169]. Immunotherapy can be administered using different types of substances. These include natural allergens, typically purified extracts, and allergoids, which are modified allergens with diminished allergenicity.

Additionally, recombinant vaccines are being developed to target specific allergens. An example of a recombinant vaccine is the PreS-Cat 1–5, which combines hypoallergenic peptides from key cat allergens (Fel d 1, Fel d 4, Fel d 7) with a carrier protein derived from the hepatitis B virus (HBV)-PreS. In preclinical studies, this vaccine demonstrated a significant reduction in IgE reactivity and allergenic activity, while also promoting the production of the tolerogenic cytokine IL-10 in cultured peripheral blood mononuclear cells [170]. These molecular vaccines appear to be highly promising, but their efficacy still needs to be validated through clinical trials.

### New Treatment Options

Strategies to reduce or eliminate animal allergens have emerged as a recent trend in research aimed at minimizing exposure for individuals who are allergic to them. One such treatment option for Fel d 1 allergic patients is a vaccine administered to cats using virus-like particles that induce strong and enduring anti-Fel d 1 antibody responses in felines. As a result of administering the vaccine to cats, antibodies are produced to neutralize the allergenic Fel d 1 molecule. Consequently, there is a decrease in the reactivity of the Fel d 1 allergen, leading to mitigation or potential prevention of allergic reactions in individuals with hypersensitivity to this allergen [171, 172]. Research conducted by Thoms further showed that individuals with allergies could interact with vaccinated cats for extended periods before experiencing allergic symptoms, resulting in a sustained reduction of such symptoms during the 2-year study period [172]. Currently, the vaccine is unavailable on the market [173].

Another promising therapeutic approach recently developed is treatment using REGN1908/1909. It is a novel cocktail of two fully human monoclonal IgG antibodies, REGN1908 and REGN1909 [174]. In a randomized, double-blind, placebo-controlled clinical trial, Shamji et al. demonstrated that a single, passive-dose administration of Fel d 1 neutralizing IgG antibodies improved the Total Nasal Symptom Score in cat-allergic patients [175]. Similarly, de Blay et al. demonstrated that a single dose of REGN1908/1909 significantly prevented drops in FEV1 in cat-allergic patients with mild asthma for up to 85 days after administration [176].

An innovative method proposed by Satyaraj et al. involves integrating anti-Fel d 1 IgY into cat nutrition to reduce the presence of active Fel d 1 in their saliva, shed hair, and dander. Consequently, this contributes to reducing active Fel d 1 in the environment and mitigates symptoms in individuals with diagnosed cat allergies [177]. Another promising recent scientific report on a novel therapeutic approach was presented at the 2023 Annual Meeting of the American Academy of Allergy, Asthma, and Immunology. In an abstract by Leyhadi et al., it has been demonstrated that a novel treatment using Fel d 1 bioparticles (BPs) could serve as an alternative to AIT. Scientists reported that Fel d 1 BP is hypoallergenic and has T and B cell tolerogenic properties [178].

Scientists and various companies are racing to deliver hypoallergenic animals to the market. However, in the most recent review exploring hypoallergenic pet options for patients allergic to furry animals in 2024, Hilger et al. concluded that there is no scientific confirmation of the presence of hypoallergenic cats or dogs on the market [179]. Furthermore, the previous consensus on cat and dog allergies, published by Dávila et al., also did not recommend “hypoallergenic” pets for individuals diagnosed with allergies to cats or dogs [180]. However, recently, a breakthrough study was published by Lee et al. Researchers utilized a Nobel-winning gene editing technique, the CRISPR-Cas9 system, and obtained Fel d 1 chain 2 (CH2) genome-edited cats, which produced significantly lower amounts of Fel d 1 than wild-type cats [181]. Nevertheless, we must wait until genetically hypoallergenic modified cats and other species become commercially available.

## Limitations

This review has several limitations that must be taken into account. First, our study focuses specifically on three groups of allergens: lipocalins, serum albumins, and secretoglobins; we did not analyze all the molecules listed in the WHO/ISUS database for furry animals. In addition, articles in English and Polish only were included in the writing of this manuscript.

Furthermore, the manuscript contains limited information on exotic animal allergen molecules and may therefore not provide an accurate picture of exotic fur animal allergy.

Finally, we have not included allergen epitope sequences; these data are publicly available on platforms such as UniProt.com and Allergome.com.

## Conclusions

Allergies to furry animals present a significant challenge, and despite recent scientific advancements, substantial gaps remain in diagnostic and treatment options. Addressing these issues is essential for enhancing the quality of life for allergic patients, particularly by implementing new therapeutic approaches that prevent progression to uncontrolled asthma.

Furthermore, developing diagnostic algorithms for the broader spectrum of allergies to furry animals is crucial, as current guidelines predominantly focus on the most common animals, such as cats, dogs, and horses. Understanding the structures of allergenic proteins across a broader range of animal species can significantly benefit a larger group of patients.

In summary, further research is needed to validate the clinical diagnostic utility of specific furry animal allergen molecules in various populations. This research would enhance the management of clinical symptoms and potential cross-reactions. Additionally, the next step may involve AIT vaccines for individuals allergic to furry animal allergens, as well as vaccinating animals to reduce the levels of allergens produced.

## Data Availability

The data used to construct Table 1 were extracted from the publication titled"A robust method for the estimation and visualization of IgE cross-reactivity likelihood between allergens belonging to the same protein family"by Chruszcz et al., 2018, available at https://doi.org/10.1371/journal.pone.0208276. The dataset presented in that study is publicly accessible and was not generated as part of the current research. It was used solely for the purpose of summarizing and visualizing previously reported findings. Appropriate credit has been given to the original authors, and the use of this data complies with the terms of the original publication.
